# Virulence of 32 *Salmonella* Strains in Mice

**DOI:** 10.1371/journal.pone.0036043

**Published:** 2012-04-27

**Authors:** Matthew C. Swearingen, Steffen Porwollik, Prerak T. Desai, Michael McClelland, Brian M. M. Ahmer

**Affiliations:** 1 Department of Microbiology, The Ohio State University, Columbus, Ohio, United States of America; 2 Vaccine Research Institute of San Diego, San Diego, California, United States of America; 3 Department of Pathology and Laboratory Medicine, University of California Irvine, Irvine, California, United States of America; Indian Institute of Science, India

## Abstract

Virulence and persistence in the BALB/c mouse gut was tested for 32 strains of *Salmonella enterica* for which genome sequencing is complete or underway, including 17 serovars within subspecies I (*enterica*), and two representatives of each of the other five subspecies. Only serovar Paratyphi C strain BAA1715 and serovar Typhimurium strain 14028 were fully virulent in mice. Three divergent atypical Enteritidis strains were not virulent in BALB/c, but two efficiently persisted. Most of the other strains in all six subspecies persisted in the mouse intestinal tract for several weeks in multiple repeat experiments although the frequency and level of persistence varied considerably. Strains with heavily degraded genomes persisted very poorly, if at all. None of the strains tested provided immunity to Typhimurium infection. These data greatly expand on the known significant strain-to-strain variation in mouse virulence and highlight the need for comparative genomic and phenotypic studies.

## Introduction

**Figure 1:**
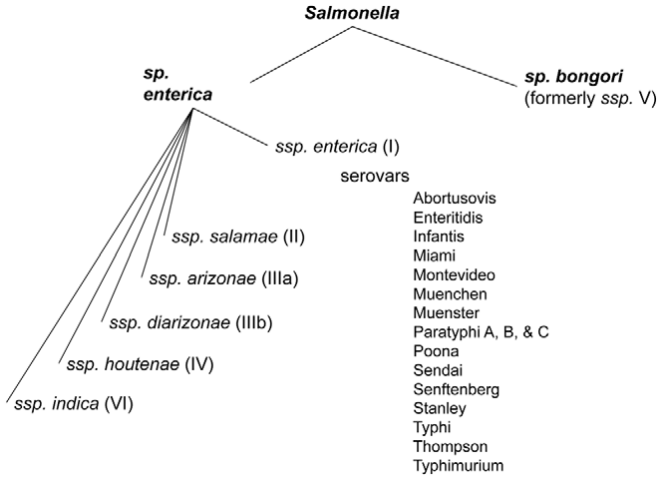
The *Salmonella* groups tested for virulence in the BALB/c mouse model. Representatives of each of the six subspecies of *Salmonella enterica* and 17 serovars within subspecies *enterica* were tested for virulence in mice. *Salmonella bongori* was not tested.

*Salmonella* is a pathogen of worldwide importance, causing disease in a vast range of hosts including humans. There are two species of *Salmonella, S. bongori* and *S. enterica. S. enterica* is comprised of six subspecies and over 2500 serovars [1] (Figure 1). There are over 1500 serovars within *S. enterica* subspecies *enterica* (also known as subspecies I) which cause ninety-nine percent of *Salmonella* infections in humans [2]. Some serovars within this subspecies such as Typhi and Paratyphi A, B, and C cause typhoid and paratyphoid fever, respectively, in humans. Symptoms of typhoid fever include headache, low to high-grade fever, nausea, lethargy, myalgia, cough, and weight loss [3]. The symptoms of paratyphoid fever may be indistinguishable from typhoid fever, except that they tend to be milder [3]. Other serovars such as Typhimurium and Enteritidis cause a self-limiting enteritis as well as more severe disease and even death in young children, the elderly or people with other diseases such as AIDS or malaria [4], [5]. The other five subspecies of *S. enterica* are more commonly associated with reptiles and amphibians and rare cases of human infection are often associated with eating or keeping reptiles as pets (Reviewed in [6]–[8]). The advent of comparative genomics has provided a new method of identifying genes involved in host range and pathogenesis. The first step towards this goal is to determine the spectrum of phenotypes of *Salmonella* in host infection. The genomes of hundreds of different isolates of *Salmonella enterica* are being sequenced. Among strains with completed and near completed genomes are two or more representatives of all six subspecies of *Salmonella enterica*.

To assay *Salmonella* strains for virulence we chose as our initial model a host that was likely to be susceptible to *Salmonella* infection. Some mouse strains, those mutated at the *slc11A1* locus (formerly known as *Nramp1*) such as BALB/c and C57/BL6 [9], present with a typhoid-like disease when infected with Typhimurium and some other serovars. Mouse lines that are *slc11A1+* are typically resistant to infection, but have proven to be important for investigating long-term persistence in the mouse, both in the intestine and the gallbladder [10]–[18]. Persistence is relevant because it occurs widely among salmonellae in a variety of animals, and it propagates the fecal-oral route of transmission. In this report, 32 *Salmonella* strains with completed or nearly completed genome sequences were screened for virulence and persistence in BALB/c mice. This work will facilitate future comparative genomic studies between the subspecies and serovars of *Salmonella*.

## Results

### Mouse virulence assays

Groups of BALB/c mice were orally inoculated with approximately 10^9^ colony forming units (CFU) of each *Salmonella* strain, and their health was monitored daily. The mice were euthanized if they met any early removal criteria (ERC - lethargy, hunched posture, or ruffled coat). Our laboratory strain, *S. enterica* subspecies *enterica* serovar Typhimurium strain ATCC14028 (14028), served as a virulent control. Only one other strain, serovar Paratyphi C BAA1715, was fully virulent, causing all mice to meet ERC in three separate experiments (Table S1). We calculated the oral LD_50_ for Paratyphi C strain BAA1715 in mice to be 1.6×10^5^ CFU, which is close to the oral LD_50_ that was calculated for the Typhimurium strain 14028 (4.5×10^5^). One of the strains tested, serovar Stanley, killed only one of 13 mice tested (Table S1).

### Fecal shedding and assessment of cross-protection

**Figure 2:**
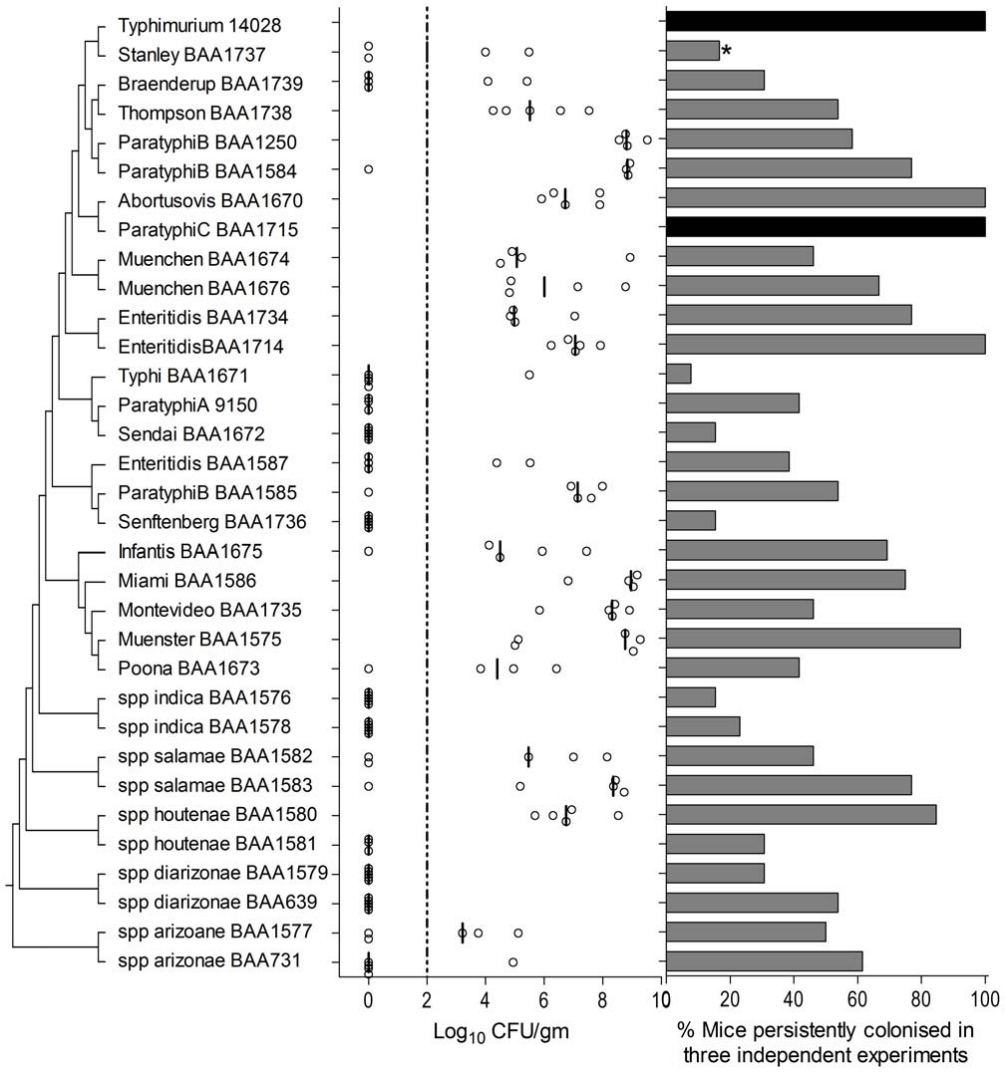
*Salmonella* recovery and persistence in the feces. The left panel shows a cladogram that was inferred from core-genome SNPS using Maximum Parsimony. All positions containing gaps and missing data were eliminated. There were a total of 344642 positions of which 279065 were parsimony informative. The parsimony analysis was conducted using Mega5 [45]. The middle panel shows *Salmonella* recovery from feces of surviving mice at 17 and 19 days post-infection. Dots represent individual measurements and the vertical line represents the median. The dotted line represents the 100 CFU/gm detection limit. The right panel represents the proportion of mice that were persistently infected with *Salmonella*. Black bars indicate that all mice met ERC. *One mouse met the ERC and was euthanized before the experiment was completed.

Only two of the 32 *Salmonella* strains studied in this report were fully mouse-virulent, but many of them were capable of colonizing the mouse intestine for several weeks. In each of the above experiments, fecal samples were collected from surviving mice toward the end of the study (between 14 and 23 days post-infection in the first experiment, at day 14 in the second experiment and between days 17 and 19 in the third experiment). In the first two experiments, the presence or absence of *Salmonella* in the feces was noted (Table S1 and Figure 2), while in the third experiment the number of *Salmonella* in the feces was quantitated (Table S1 and Figure 2). Although there was a lot of variability, the strains clearly differed in their ability to colonize the mice and to be shed in feces.

Avirulent *Salmonella* strains may exist that provide protection against virulent strains. These would be candidates for live vaccine strains. Therefore, at the end of each virulence experiment, all surviving mice were challenged intragastrically with 1×10^9^ CFU of serovar Typhimurium strain 14028. None of the *Salmonella* strains provided protection against 14028.

## Discussion

Among the 32 strains tested in this report only Typhimurium strain 14028 and Paratyphi C strain BAA1715 were fully virulent in mice. The oral LD_50_ for 14028 and BAA1715 were 4.5×10^5^ and 1.6×10^5^ CFU, respectively. While this particular strain of Paratyphi C had not been tested prior to this study, other strains of Paratyphi C have been shown to cause disease in mice [19]–[25]. However, the strains we tested from some serovars previously reported to be virulent in mice (Abortusovis, Enteritidis, and Montevideo, see references in Table S1) were not found to be virulent in this study. The reason for the avirulence of the Abortusovis and Montevideo isolates is unknown. However, two of the Enteritidis strains studied here, BAA1587 and BAA1734, were selected because their genomes differ markedly from the main Enteritidis clade, and they represent very rare genomovars of this serotype. Their inability to kill mice is therefore not surprising. The reason(s) for the discrepancies in virulence between individual strains of particular serovars or subspecies can be due to genetic variations between strains that belong to the same serotype but differ in phylogeny. Porwollik et. al. demonstrated that there can be hundreds of genes differing between isolates of a single serovar [28].

The third avirulent strain of Enteritidis, BAA1714, was the earliest isolated strain of Enteritidis available to us. This strain was isolated from a guinea pig in 1948, prior to Enteritidis becoming widespread in chickens and a danger to humans [26], [27]. Thus, it is possible that this strain has lost virulence during its 60 years of storage or is not the lineage that expanded in chickens and proved to be pathogenic to humans. In this study, serovar Stanley led to the death of one out of 13 total mice tested. Serovar Stanley has not previously been reported to be virulent in mice although it has been isolated from rats and is a human pathogen [29]–[34]. Serovars Thompson, Poona, Paratyphi A, and Infantis have never been shown to cause disease in mice, consistent with the results in this study, but they have been isolated from wild mice and rats [35], [36] and from laboratory mice [37]. Consistent with this, we observed that our isolates of Thompson, Poona, and Infantis were recovered from feces two to three weeks after inoculation. However, Paratyphi A was not. The genes required for these strains to colonize the intestinal tract are not known but could be the focus of future studies. Additionally, serovars Typhimurium, Enteritidis, Anatum and California were commonly isolated from laboratory mice in the days before Specific Pathogen Free certifications [37].

In reviewing the literature (Table S1) we found that some strains that belong to human-restricted serotypes have been isolated from peculiar places. One study reported that serovar Typhi had been isolated from camels in the United Arab Emirates and Ethiopia (none of the animals presented symptoms) [38], [39]. Another study suggested grey duiker antelope as a possible reservoir for *S.* Typhi, as individuals who worked as bushmeat processors were seropositive for *S.* Typhi. No attempt was made to isolate *S.* Typhi from the grey duiker antelope, but the animals were seropositive for *S.* Typhi [40]. In 1977, Lavergne et. al. developed an asymptomatic carrier model in guinea pigs via a surgically cannulated gall bladder, and Typhi could be isolated from the bile and feces for up to five months post-infection [41]. It was later shown that Typhi could cause systemic infection by a more natural oral infection in newborn guinea pigs [42]. While the strains of Typhi and Paratyphi A that we tested in this experiment were not virulent in mice, an older report shows that isolates of these serovars, which had been isolated from the heart blood of a dead hen and rabbit, respectively, were able to kill laboratory mice [43]. Whether or not the host-restricted strains can truly colonize these animals is not known, as there have not been repeated animal isolations or experimental confirmations. In contrast, the serovars that are considered to have a broad host-range, such as Typhimurium and Enteritidis, have been repeatedly isolated from up to forty host organisms. Table S1 exemplifies the ubiquitous nature of broad host-range *Salmonella*.

This work demonstrates that significant variations in pathogenicity can occur between strains of *Salmonella* that, according to serovar classification, are closely related. These results reinforce the need for strain genome sequencing, and suggest the need for additional genomovar classification of *Salmonella* strains. Furthermore, the fact that strains of the same serovar can vary significantly in pathogenesis within the same host highlights the possibility of identifying virulence factors using comparative genomics.

## Methods

### Ethics statement

This study was performed in strict accordance with animal use protocols approved by The Ohio State University Institutional Animal Care and Use Committee (IACUC, protocol number OSU 2009A0035). Mice were euthanized if they met any early removal criteria (lethargy, hunched posture, or ruffled coat) to limit suffering.

### Bacterial strains and media

All *Salmonella* strains used in this study are listed in Table 1. Salmonellae were grown with shaking in Luria-Bertani (LB) broth at 37°C (EMD Chemicals, Gibbstown, NJ).

### Mouse virulence and fecal shedding

Female BALB/c mice (8 to 10 weeks old) were obtained from Harlan laboratories. Overnight cultures of each *Salmonella* strain were centrifuged at 5000×g and resuspended in fresh LB broth and kept on melting ice. Mice were inoculated intragastrically with 0.2 ml of each *Salmonella* strain (approximately 10^9^ total CFU), and dilution plating of each inoculum was used to determine the actual dose administered.

Xylose-lysine-desoxycholate (XLD) agar (EMD Chemicals) plates were used for the recovery of *Salmonella* from feces. Fecal pellets from surviving mice were homogenized and dilution plated for enumeration. Surviving mice were challenged with 10^9^ CFU of 14028 to assess if immunity was elicited by any test strains.

### LD_50_ determinations

Inocula of 14028 and Paratyphi C strain BAA1715 were prepared as described above. The suspensions were serially diluted in LB broth and groups of five mice were inoculated with doses ranging from 10^1^ to 10^9^ CFU. Mice meeting ERC were euthanized. The LD_50_ was calculated using the method of Reed and Muench [44].

## Supporting Information

Table S1 **^a^ Current American Type Culture Collection (ATCC) strain numbers, ^b^ subspecies or serovar name, ^c^ specific strain collections if applicable, ^d^ number of mice meeting ERC out of the total tested in three different experiments, ^e^ number of mice that had** ***Salmonella*** **in feces between 14 and 23 days post-infection in the first experiment, at day 14 in the second experiment and between days 17 and 19 in the third experiment.** In the first two experiments the presence/absence call had a detection limit of 100 CFU, ^f^ information about sources, antigenic formulae, electrophoretic types, and strain aliases, and ^g^ a literature review of the animal sources or models for the salmonellae tested. Abbreviations: *Salmonella* genetic stock center (SGSC), *Salmonella* reference collection B and C (SARB and SARC, respectively), year (yr.) years old (y), accession number (Acc:), chromosome (chro), plasmid (plsm), and Center for Disease Control (CDC). * denotes virulence; ** denotes laboratory model; *** denotes isolation but not necessarily virulence, ****denotes seropositive.
(DOCX)

## References

[1] Guibourdenche M, Roggentin P, Mikoleit M, Fields PI, Bockemuhl J (2009). Supplement 2003–2007 (No. 47) to the White-Kauffmann-Le Minor scheme.. Res Microbiol.

[2] Lan R, Reeves PR, Octavia S (2009). Population structure, origins and evolution of major Salmonella enterica clones.. Infect Genet Evol.

[3] Bhan MK, Bahl R, Bhatnagar S (2005). Typhoid and paratyphoid fever.. Lancet.

[4] MacLennan CA, Gilchrist JJ, Gordon MA, Cunningham AF, Cobbold M (2011). Dysregulated humoral immunity to nontyphoidal Salmonella in HIV-infected African adults.. Science.

[5] Roux CM, Butler BP, Chau JY, Paixao TA, Cheung KW (2010). Both hemolytic anemia and malaria parasite-specific factors increase susceptibility to Nontyphoidal Salmonella enterica serovar typhimurium infection in mice.. Infect Immun.

[6] Magnino S, Colin P, Dei-Cas E, Madsen M, McLauchlin J (2009). Biological risks associated with consumption of reptile products.. Int J Food Microbiol.

[7] Enriquez C, Nwachuku N, Gerba CP (2001). Direct exposure to animal enteric pathogens.. Rev Environ Health.

[8] Warwick C, Lambiris AJ, Westwood D, Steedman C (2001). Reptile-related salmonellosis.. J R Soc Med.

[9] Nairz M, Fritsche G, Crouch ML, Barton HC, Fang FC (2009). Slc11a1 limits intracellular growth of Salmonella enterica sv. Typhimurium by promoting macrophage immune effector functions and impairing bacterial iron acquisition.. Cell Microbiol.

[10] Monack DM, Bouley DM, Falkow S (2004). Salmonella typhimurium persists within macrophages in the mesenteric lymph nodes of chronically infected Nramp1+/+ mice and can be reactivated by IFNgamma neutralization.. J Exp Med.

[11] Kingsley RA, Humphries AD, Weening EH, De Zoete MR, Winter S (2003). Molecular and phenotypic analysis of the CS54 island of Salmonella enterica serotype typhimurium: identification of intestinal colonization and persistence determinants.. Infect Immun.

[12] Weening EH, Barker JD, Laarakker MC, Humphries AD, Tsolis RM (2005). The Salmonella enterica serotype Typhimurium lpf, bcf, stb, stc, std, and sth fimbrial operons are required for intestinal persistence in mice.. Infect Immun.

[13] van der Woude MW, Baumler AJ (2004). Phase and antigenic variation in bacteria.. Clin Microbiol Rev.

[14] Crawford RW, Gibson DL, Kay WW, Gunn JS (2008). Identification of a bile-induced exopolysaccharide required for Salmonella biofilm formation on gallstone surfaces.. Infect Immun.

[15] Crawford RW, Reeve KE, Gunn JS (2010). Flagellated but not hyperfimbriated Salmonella enterica serovar Typhimurium attaches to and forms biofilms on cholesterol-coated surfaces.. J Bacteriol.

[16] Crawford RW, Rosales-Reyes R, Ramirez-Aguilar Mde L, Chapa-Azuela O, Alpuche-Aranda C (2010). Gallstones play a significant role in Salmonella spp. gallbladder colonization and carriage.. Proc Natl Acad Sci U S A.

[17] Monack DM (2011). Salmonella persistence and transmission strategies.. Curr Opin Microbiol.

[18] Gonzalez-Escobedo G, Marshall JM, Gunn JS (2011). Chronic and acute infection of the gall bladder by Salmonella Typhi: understanding the carrier state.. Nat Rev Microbiol.

[19] Estevan M, Irache JM, Grillo MJ, Blasco JM, Gamazo C (2006). Encapsulation of antigenic extracts of Salmonella enterica serovar. Abortusovis into polymeric systems and efficacy as vaccines in mice.. Vet Microbiol.

[20] Uzzau S, Marogna G, Leori GS, Curtiss R, Schianchi G (2005). Virulence attenuation and live vaccine potential of aroA, crp cdt cya, and plasmid-cured mutants of Salmonella enterica serovar Abortusovis in mice and sheep.. Infect Immun.

[21] Mukkur TK, Stocker BA, Walker KH (1991). Genetic manipulation of Salmonella serotype Bovismorbificans to aromatic-dependence and evaluation of its vaccine potential in mice.. J Med Microbiol.

[22] Collins FM (1968). Cross-protection against Salmonella enteritidis infection in mice.. J Bacteriol.

[23] Archer GT, Whitby JL (1957). Protection of mice by living Vi and O vaccines against death caused by Salmonella paratyphi C.. J Hyg (Lond).

[24] Barber C, Eylan E (1975). Heterologous protections in experimental salmonellosis.. Zentralbl Bakteriol Orig A.

[25] Howard JG (1961). Resistance to infection with Salmonella paratyphi C in mice parasitized with a relatively avirulent strain of Salmonella typhimurium.. Nature.

[26] Boyd EF, Wang FS, Beltran P, Plock SA, Nelson K (1993). Salmonella reference collection B (SARB): strains of 37 serovars of subspecies I.. J Gen Microbiol.

[27] Beltran P, Plock SA, Smith NH, Whittam TS, Old DC (1991). Reference collection of strains of the Salmonella typhimurium complex from natural populations.. J Gen Microbiol.

[28] Porta A, Eletto A, Torok Z, Franceschelli S, Glatz A (2010). Changes in membrane fluid state and heat shock response cause attenuation of virulence.. J Bacteriol.

[29] Joseph PG, Yee HT, Sivanandan SP (1984). The occurrence of Salmonellae in house shrews and rats in Ipoh, Malaysia.. Southeast Asian J Trop Med Public Health.

[30] Lee JA (1974). Recent trends in human salmonellosis in England and Wales: the epidemiology of prevalent serotypes other than Salmonella typhimurium.. J Hyg (Lond).

[31] Besznyak I, Pinter E, Turbok E (1965). Thoracic empyema and lung abscess due to Salmonella stanley.. Arch Surg.

[32] Ray K, Aggarwal P, Rai Chowdhuri AN (1983). Simultaneous isolation of Salmonella stanley and S. oranienburg from an outbreak of food poisoning at Maldives island.. Indian J Med Res.

[33] Angkititrakul S, Chomvarin C, Chaita T, Kanistanon K, Waethewutajarn S (2005). Epidemiology of antimicrobial resistance in Salmonella isolated from pork, chicken meat and humans in Thailand.. Southeast Asian J Trop Med Public Health.

[34] Lazarus R, Waghorn D, Nash C (2007). Cutaneous Salmonella infection.. Scand J Infect Dis.

[35] Shimi A, Keyhani M, Hedayati K (1979). Studies on salmonellosis in the house mouse, Mus musculus.. Lab Anim.

[36] Franklin J, Richter CB (1968). The isolation of Salmonella poona and a nonmotile variant from laboratory mice.. Lab Anim Care.

[37] Margard WL, Litchfield JH (1963). Occurrence of Unusual Salmonellae in Laboratory Mice.. J Bacteriol.

[38] Wernery U (1992). The prevalence of Salmonella infections in camels (Camelus dromedarius) in the United Arab Emirates.. Br Vet J.

[39] Molla B, Mohammed A, Salah W (2004). Salmonella prevalence and distribution of serotypes in apparently healthy slaughtered camels (Camelus dromedarius) in Eastern Ethiopia.. Trop Anim Health Prod.

[40] Ogunsanmi AO, Taiwo VO, Iroeche PC, Sobaloju SO (2001). Serological survey of salmonellosis in grey duiker (Sylvicapra grimmia) in Asejire, Irewole Local Government Area, Osun State, Nigeria.. Afr J Med Med Sci.

[41] Lavergne GM, James HF, Martineau C, Diena BB, Lior H (1977). The guinea pig as a model for the asymptomatic human typhoid carrier.. Lab Anim Sci.

[42] Dima VF, Petrovici M, Lacky D (1989). Reaction and response of newborn guinea pigs to experimental Salmonella typhi infection.. Arch Roum Pathol Exp Microbiol.

[43] Kulkarni VB, Narasimham AS (1966). Isolation of unusual Salmonella types (S. typhi and S. paratyphi A) from a hen and rabbit respectively.. Indian J Pathol Bacteriol.

[44] Reed LJ, Muench H (1938). A simple method for estimating fifty percent endpoints.. American Journal of Hygiene.

[45] Tamura K, Peterson D, Peterson N, Stecher G, Nei M (2011). MEGA5: molecular evolutionary genetics analysis using maximum likelihood, evolutionary distance, and maximum parsimony methods.. Mol Biol Evol.

