# Supplementary material for: Virulence of 32 Salmonella Strains in Mice
Source: PLoS One. 2012 Apr 27;7(4):e36043. doi: 10.1371/journal.pone.0036043 (PMC3338620; doi:10.1371/journal.pone.0036043)
Supplement: Table S1 — a Current American Type Culture Collection (ATCC) strain numbers, b subspecies or serovar name, c specific strain collections if applicable, d number of mice meeting ERC out of the total tested in three different experiments, e number of mice that had Salmonella in feces between 14 and 23 days post-infection in the first experiment, at day 14 in the second experiment and between days 17 and 19 in the third experiment. In the first two experiments the presence/absence call had a detection limit of 100 CFU, f information about sources, antigenic formulae, electrophoretic types, and strain aliases, and g a literature review of the animal sources or models for the salmonellae tested. Abbreviations: Salmonella genetic stock center (SGSC), Salmonella reference collection B and C (SARB and SARC, respectively), year (yr.) years old (y), accession number (Acc:), chromosome (chro), plasmid (plsm), and Center for Disease Control (CDC). * denotes virulence; ** denotes laboratory model; *** denotes isolation but not necessarily virulence, ****denotes seropositive. (DOCX) [file pone.0036043.s001.docx]

Table S1

| ^a^ ATCC# | ^b^ Description | ^c^ Name | ^d^ Mice that met ERC | ^e^ Mice that shed *Salmonella* in feces | ^f^ Notes | ^g^ Source reference |
| --- | --- | --- | --- | --- | --- | --- |
| BAA1670 | Abortusovis | SSM0041 | 0/5, 0/5, 0/5 | 5/5, 5/5, 5/5 | Source: Sergio Uzzau, Italy; isolated in USSR before 1990; no IS1414 | Sheep [20*,46*], Goat [46]*, cattle [46]*, mice [19,20]* |
| BAA1577 | ssp. arizonae | 05-0715 | 0/4, 0/3, 0/5 | 0/4, 3/3, 3/5 | Source: SGSC; from SARC collection; Serotype 62:z4,z23:-; original strain name: CDC 346-86, other strain names: RKSs2980 & SGSC3061; isolated in 1986 from corn snake in Oregon, USA. Acc: chro [NC_010067.1](http://www.ncbi.nlm.nih.gov/nuccore/161501984) | human [47-49]*, cat [50]*, snake [51***,52**], rat snake, boa [53]***, pigs [54]***, boar [55]***, cattle [54***,56*,57***], buffalo [54]***, turtle [58,59]***, lizard [60]***, chicken [61]*, lambs [62]***, turkey vulture [63]***, turkey [64]*, horse [65]*, sheep [66**,67***], croccodile [68]***, rhinoceros [69]*, lynx [70]*, cockatoo [71]*, iguana [71]***, gecko [72]*** |
| BAA731 | ssp. arizonae | SARC05 | 0/5, 0/3, 0/5 | 5/5, 2/3, 1/5 | Source: SGSC; SGSC 4074 | see above |
| BAA1739 | Braenderup | S-500 | 0/5, 0/3, 0/5 | 0/5, 2/3, 2/5 | Source:  CDC; serotype 61:1,v:1,5 | humans [73,74]*, cockroaches [75]***, chicken and duck [54]***, turtle [76*,77***], camel [39]***, cattle and birds [78]***, pigs [79,80]***, quail [81]**, eagle [82]***, oppossum [83]*, squirrel, woochuck, hawk [83]*, owl [83]*/*** |
| BAA639 | ssp. diarizonae | 01-005 | 0/5, 0/3, 0/5 | 4/5, 3/3, 0/5 | Source: CDC; serotype 48:i:z | human [84]*, crocodiles [85]***, snakes [86]***, California King Snake, Common egg-eater Dasypeltis scabra, water dragon, veiled chameleon, chameleon, and frillneck lizard [53]***, ram [87]* |
| BAA1579 | ssp. diarizonae | 05-0625 | 0/5, 0/3, 0/5 | 4/5, 0/3, 0/5 | Source: SGSC; from SARB collection, original strain name: IVB 176/82, other strain names: RKS761 & SGSC2474; isolated in Brazil | see above |
| BAA1587 | Enteritidis | SARB17 | 0/5, 0/3, 0/5 | 0/5, 3/3, 2/5 | Source: CDC; old *Salmonella* strain isolated from a guinea pig (NIH) (yr. 1948) | human [88-90]*, reptiles [53]***, mice [22,91,92]*, cats [93*,94***], dog [89,95]***, chicken [96,97]***, geese [96]***, hens [96]***, rat [98,99]*, cattle [78,100]*, camel [38,39]***, horse [101]*, deer [77*,102***], turkey [103]***, pig [78,97]*, ducks [73]*, owl, fox [55]***, poultry and birds [78]***, sheep [78]*, mink [104]***, house fly [105]***, crocodile [68]***, dogs [54]***, stellar sea lion pups [106]*** |
| BAA1714 | Enteritidis | 48-0811 | 0/5, 0/5, 0/5 | 5/5, 5/5, 5/5 | Source: SGSC; from SARB collection; electrophoretic type En7; original strain name: IVB 470/82, other strain names: RKS1208 & SGSC2476; isolated in Switzerland | see above |
| BAA1734 | Enteritidis | SARB19 | 0/5, 0/3, 0/5 | 5/5, 1/3, 4/5 | Source: CDC; serotype 48:g,z51:-. Acc: [AGRM00000000.1](http://www.ncbi.nlm.nih.gov/nuccore/379053785) | see above |
| BAA1581 | ssp. houtenae | 05-0642 | 0/5, 0/3, 0/5 | 1/5, 3/3, 0/5 | Source: CDC; serotype 50:z4,z23:- | human [107-109]*, pigs, chicken, ducks [54]***, chameleon [110]***, cockateel [111]*, oppossum [112]*, tiger python, Common egg-eater Dasypeltis scabra, bearded dragon [53]***, iguana [53***, 110***] |
| BAA1580 | ssp. houtenae | 99-0125 | 0/5, 0/3, 0/5 | 4/5, 2/3, 5/5 | Source: SGSC; SARC collection; serotype 45:a:e,n,x; original strain name: CDC 1363-65, other strain names: RKSs2995 & SGSC3116; isolated in India (yr. 1965) | see above |
| BAA1578 | ssp. indica | SARC13 | 0/5, 0/3, 0/5 | 3/5, 0/3, 0/5 | Source: SGSC; SARC collection; serotype 11:b:e,n,x; original strain name: CDC 347-78, other strain names: RKSs3057 & SGSC3118; isolated (yr. 1978) | human [113]* |
| BAA1576 | ssp. indica | SARC14 | 0/5, 0/3, 0/5 | 0/5, 2/3, 0/5 | Source: SGSC; from SARB collection; serotype 6,7:r:1,5; electrophoretic type In3;  Biotype not given; original strain name: IVB 385/72, other strain names: RKS1452 & SGSC2484; isolated in Senegal. Acc: chro [NZ_AFYI00000000.1](http://www.ncbi.nlm.nih.gov/nuccore/375004549) | see above |
| BAA1675 | Infantis | SARB27 | 0/5, 0/3, 0/5 | 2/5, 3/3, 4/5 | Source: CDC; originated from New Mexico; serotype 9,12:a1,5  from male human stool (36 y) | human [73,88,90,114]*, pigs [115***,116*,117***], cattle [73***,97***,100*,118*], bovine [54]***, poultry [54,119-121]***, horse [101***,122*], turkey [103***,123*], mink [104]***, freshwater snail [124]***, double-crested commorant [125]***, dog [126]*, house fly [105]***, deer [102]***, rat [99]***, quail [81]*/**, iguana [53]***, mice [37]***, birds,poultry, pigs [78]*, stellar sea lion pups [106]*** |
| BAA1586 | Miami | 02-0341 | 0/5, 0/3, 0/5 | 2/5, 3/3, 4/4 | Source: SGSC; from SARB collection; Serotype 6,7:g,m,[p],s:[1,2,7]; electrophoretic type Mo1; Biotype not given; original strain name: CDC B2131, other strain names: RKS1762 & SGSC2487; isolated from human in Georgia. Acc: [AESU00000000.1](http://www.ncbi.nlm.nih.gov/nuccore/363569422) | human [26,127]*, snake [77*,128***], lizard [128]*** |
| BAA1735 | Montevideo | SARB30 | 0/5, 0/3, 0/5 | 1/5, 0/3, 5/5 | Source: SGSC; from SARB collection; serotype 6,8:d:1,2; electrophoretic type Mu3;  biotype not given; original strain name: IP25/88, other strain names: RKS4300 & SGSC2491; isolated from human in France (yr. 1988) | human [73,129-131]*, chicken/ducks [54]***, cattle [97***,132***,133*], mice [37***,134*], sheep [135,136]*, birds and poultry [78***,103***137***,138*], pigs [78]***, horse [122]*, herring gull [139]***, snake [140]***, foxhound [141]*, dog [142]*, ewe [141]*, sea gulls [143]***, salmon [144]**, bearded dragon [53]***, monitor [53]*** |
| BAA1674 | Muenchen | SARB34 | 0/5, 0/3, 0/5 | 0/5, 2/3, 4/5 | Source: SGSC; from SARB collection; serotype 6,8:d:1,2; electrophoretic type Mu1;  biotype not given; original strain name: ATCC8388, other strain names: RKS3121 & SGSC2489; laboratory strain | human [27,73,145]*, cattle [146]***, pigs [116**,147***], camel [38,39]***, iguana [110]***, turtle [148***,149**], lizard [110]***, elk [73]***, mice [35]***, goat [150]*, chicken, cattle [27]***, cheetah [151]*, hawk [55]***, birds and poultry [78]***, western grey kangaroos [152]*** |
| BAA1676 | Muenchen | SARB32 | 0/5, 0/3, 0/5 | 2/5, 2/3, 4/4 | Source: CDC; isolated from female human stool (23 y) | see above |
| BAA1575 | Muenster | 0065-00 | 0/5, 0/3, 0/5 | 4/5, 3/3, 5/5 | Source: SGSC; from SARB collection; serotype 1,2,12:a[1,5]; electrophoretic type Pa1; Biotype not given; other strain names: RKS4993 & SGSC2499; laboratory strain. Acc: chro [NC_006511.1](http://www.ncbi.nlm.nih.gov/nuccore/56412276) | human [153]*, pig [154]***, cattle [78*,132***,133*,155], turkey [97,103,156]***, sheep [78]*, birds and poultry [78*,137***] |
| 9150 | Paratyphi A | SARB42 | 0/5, 0/3, 0/5 | 5/5, 0/3, 0/4 | Source: SGSC; strain SPB7; isolated in Penang, Malaysia, (yr. 2002) from human stool; Acc: chro [NC_010102.1](http://www.ncbi.nlm.nih.gov/nuccore/161612313) | human [157-159]*, rabbit [43*,160**], cattle [159]***, hamster [161]**, lamb [43]*, mice [37]***, hogs [43]***, rat [43]*** |
| BAA1250 | Paratyphi B | SGSC4150 | 0/5, 0/3, 0/5 | 0/5, 3/3, 4/4 | Source: SGSC; serotype 1,4,[5],12:b:[1,2]; electrophoretic type Pb7; Biotype 1g; original strain name: DMS53/81, other strain names: RKS3215 & SGSC2504; isolated from human in Africa (yr. 1981) | human [158,162-166]*, horse [167]*, mice [168]**, cattle [169-171*], goats [54]***, poultry [172**,173***], turtle [174]***, darkling beetle [175]**, rat [176]***, guineapigs [177]*, dog [54,178]***, pigs [54]***, snake [77]***, cats [54]***, shrew [29]***, grey duiker antelope [40]**** |
| BAA1585 | Paratyphi B | SARB47 | 0/5, 0/3, 0/5 | 0/5, 3/3, 4/5 | Source: SGSC (SGSC 4081) | see above |
| BAA1584 | Paratyphi B | S-1241 | 0/5, 0/3, 0/5 | 4/5, 3/3, 3/4 | Source: SGSC; from SARB collection; serotype 6,7[Vi]:c:1,5; electrophoretic type Pc2; original strain name: IP2/88, other strain names: RKS4594 & SGSC2506; isolated from human in France (yr 1988). Acc: chro [NC_012125.1](http://www.ncbi.nlm.nih.gov/nuccore/224581838), plsm [NC_012124.1](http://www.ncbi.nlm.nih.gov/nuccore/224504235) | see above |
| BAA1715 | Paratyphi C | SARB49 | 5/5, 3/3, 5/5 | N/A | Source: Casey Poppe, University of Guelph, CANADA; strain# SA20023503; isolated from iguana intestine in Ontario, CA (yr. 1997) | humans [179-181]*, mice [23,24]**, grey duiker antelope [40]**** |
| BAA1673 | Poona | SGSC4934 | 0/5, 0/3, 0/5 | 1/5, 1/3, 3/4 | Source: CDC, serotype 47:b:1,5 | human [182,183]*, iguana [110,184]***, turtle [58,59,110]***, lizard [110]***, goats [185]*, sheep [78,186]***, great cane rat [187]***, C. elegans [188,189]**, guineapigs, dog, cat, pig, birds, mice [36]*** |
| BAA1583 | ssp. salamae | 05-0626 | 0/5, 0/3, 0/5 | 4/5, 2/3, 4/5 | Source: SGSC, SARC collection; serotype: 58:d:z6; original strain name: CDC 151-85, other strain names: RKSs2985 & SGSC3039; isolated from human in Massachussetts, USA (yr. 1985) | crocodiles [85]***, sheep [190]***, turtle [191]***, leopard gecko [53]*** |
| BAA1582 | ssp. salamae | SARC03 | 0/5, 0/3, 0/5 | 0/5, 3/3, 3/5 | Source: CDC; isolated in England; serotype 9,12:a1,5; aka CDC Stk.475 | see above |
| BAA1672 | Sendai | 55-2461 | 0/5, 0/3, 0/5 | 0/5, 2/3, 0/5 | Source: SGSC; from SARB collection; serotype 1,3,19:g,[s],t:- -; electrophoretic type Sf1; Biotype not given; original strain name: NVSL6673, other strain names: RKS2358 & SGSC2516; isolated from chicken in Maryland, USA (yr. 1987) | human [192,193]* |
| BAA1736 | Senftenberg | SARB59 | 0/5, 0/3, 0/5 | 0/5, 2/3, 0/5 | Source: SGSC; from SARB collection; serotype 1,4,[5],12,27:d: 1,2; electrophoretic type St1; Biotype 26bei; original strain name: DMS1112, other strain names: RKS4264 & SGSC2517; isolated in Scotland (yr. 1988) | human [194]*, cattle [54,78]***, buffalo [54]***, chicken [26,54]***, ducks [54]***, turkey [103,156]***, birds and poultry [78,119]***, sea gulls [143]***, pigs [80]***, mink [104]***, oppossum, squirrel, woodchuck, hawk, owl [83]*** |
| BAA1737 | Stanley | SARB60 | 1/5, 0/3, 0/5 | 0/4, 0/3, 2/4 | Source: SGSC; from SARB collection; serotype 6,7:k:1,5; electrophoretic type Th1; Biotype not given; original strain name: CDC B2637, other strain names: RKS1767 & SGSC2519; isolated from human in Florida, USA | human, [30-34]*, pig [30,33,54,195]***, poultry [30,54]***, sea gulls [196]***, monkey [54***,197*], turtle [110]***, cattle [198]***, shrews, rats, dogs, guinea pigs, shrew [29]***, stellar sea lion pups [106]*** |
| BAA1738 | Thompson | SARB62 | 0/5, 0/3, 0/5 | 0/5, 2/3, 5/5 | Source: SGSC; strain # CDC1707-81; isolated in Liberia; O group D; antigenic formula 9,12,Vi:d:-; Phage type UT (Vi - neg); ET 1 | human [26,199]*, chicken [200*,201***], ducks [54]***, cattle [201***,202*], pigs [78,97]***, landfowl [203]*, mice [35]*, owl [204]***, horse [101,201]***, sheep [78,201]**, dog, snake [201]***, rodent, waterbird, eagle [77]***, turtle [205]***, turkey [97]*** |
| BAA1671 | Typhi | SGSC2661 | 0/5, 0/3, 0/5 | 0/5, 0/3, 1/5 | Source: ATCC strain, most common virulent Typhimurium strain used in laboratories, Designations: CDC 6516-60 [4016, CIP 104115, NCTC 12023], Isolation: tissue, animal (pools of heart and liver from 4-week-old chickens) at CDC University of Missouri, USA. Acc: [NC_016856.1](http://www.ncbi.nlm.nih.gov/nuccore/378448274), plsm [NC_016855.1](http://www.ncbi.nlm.nih.gov/nuccore/378448040) | human [206,207]*, hen [43]*, guineapigs [41]**, newborn guineapigs [42]**, camel [38]***, mice [43,168]**, grey duiker antelope [40]**** |
| 14028 | Typhimurium |  | 5/5, 3/3, 5/5 | N/A |  | human [208], rhesus macaques [209]**, mice [208,210,211]**, horse [167***,173***,212*], camel [38,39]***, frog [213]***, cat [93***,94***,214*], cockroaches [75]***, sheep [215**,216*], geese [96]***, chicken [96***,217**], helminth [218]**, chicken egg [219]***, cattle [220**,221**,222***], *C. elegans* [223,224]**, pig [116]*, pigmy hogs [225]*, guineapig [177*,226**], parakeet [214]*, rat [227]**, zebra finch [228]***, feeder bird [229]***, bobwhite quail [230]*, sika deer [231]*, ostrich [232]*, hedgehogs [233,110]***, owl [234]***, fox [235]*, parrot, heron [26,27]***, porcupine [77]***, snake [77***,52**], turkey [103,73]***, bison [73]***, goat [150]*, rabbit, oppossum [27]***, dog [27***,95*], cheetah [151]*, mink [104]*, crow [125]***, double-crested commorant [125]***, black marsh turtle [53]*** |

Supporting Refernces

46. Pioz M, Loison A, Gauthier D, Gibert P, Jullien JM, et al. (2008) Diseases and reproductive success in a wild mammal: example in the alpine chamois. Oecologia 155: 691-704.
47. Arora S, Tyagi SC (1976) Bacteremia due to Salmonella arizonae. J Assoc Physicians India 24: 457-458.
48. Kaura YK, Sharma VK, Chandiramani NK (1982) Enterotoxigenicity and invasiveness of Salmonella species. Antonie Van Leeuwenhoek 48: 273-283.
49. Bhatt BD, Zuckerman MJ, Foland JA, Polly SM, Marwah RK (1989) Disseminated Salmonella arizona infection associated with rattlesnake meat ingestion. Am J Gastroenterol 84: 433-435.
50. Krum SH, Stevens DR, Hirsh DC (1977) Salmonella arizonae bacteremia in a cat. J Am Vet Med Assoc 170: 42-44.
51. Cahan D, Sechter I, Gerichter CB, Greenberg Z, Sklut O, et al. (1977) Six new Salmonella sub-genus III serotypes isolated from snakes in Israel. Ann Microbiol (Paris) 128A: 69-73.
52. Chiodini RJ (1982) Transovarian passage, visceral distribution, and pathogenicity of salmonella in snakes. Infect Immun 36: 710-713.
53. Pedersen K, Lassen-Nielsen AM, Nordentoft S, Hammer AS (2009) Serovars of Salmonella from captive reptiles. Zoonoses Public Health 56: 238-242.
54. Joseph PG, Sivanandan SP, Yee HT (1988) Animal Salmonella surveillance in Peninsular Malaysia, 1981-1985. Epidemiol Infect 100: 351-359.
55. Millan J, Aduriz G, Moreno B, Juste RA, Barral M (2004) Salmonella isolates from wild birds and mammals in the Basque Country (Spain). Rev Sci Tech 23: 905-911.
56. Gaspar P (1978) Isolation of Salmonella arizonae from an aborted bovine foetus. Bull Anim Health Prod Afr 26: 230-231.
57. Rodriguez A, Pangloli P, Richards HA, Mount JR, Draughon FA (2006) Prevalence of Salmonella in diverse environmental farm samples. J Food Prot 69: 2576-2580.
58. Shane SM, Gilbert R, Harrington KS (1990) Salmonella colonization in commercial pet turtles (Pseudemys scripta elegans). Epidemiol Infect 105: 307-316.
59. D'Aoust JY, Daley E, Crozier M, Sewell AM (1990) Pet turtles: a continuing international threat to public health. Am J Epidemiol 132: 233-238.
60. Mathewson JJ (1979) Enterobacteriaceae isolated from iguanid lizards of west-central Texas. Appl Environ Microbiol 38: 402-405.
61. Silva EN, Hipolito O, Grecchi R (1980) Natural and experimental Salmonella arizonae 18:z4,z32 (Ar. 7:1,7,8) infection in broilers. Bacteriological and histopathological survey of eye and brain lesions. Avian Dis 24: 631-636.
62. Harp JA, Myers LL, Rich JE, Gates NL (1981) Role of Salmonella arizonae and other infective agents in enteric disease of lambs. Am J Vet Res 42: 596-599.
63. Winsor DK, Bloebaum AP, Mathewson JJ (1981) Gram-negative, aerobic, enteric pathogens among intestinal microflora of wild turkey vultures (Cathartes aura) in west central Texas. Appl Environ Microbiol 42: 1123-1124.
64. Jortner BS, Larsen C (1984) Granulomatous ventriculitis of the brain in arizonosis of turkeys. Vet Pathol 21: 114-115.
65. Adamson PJ, Jang SS (1985) Ulcerative keratitis associated with Salmonella arizonae infection in a horse. J Am Vet Med Assoc 186: 1219-1220.
66. Hannam DA, Wray C, Harbourne JF (1986) Experimental Salmonella arizonae infection of sheep. Br Vet J 142: 458-466.
67. Pritchard J (1990) Alberta. Salmonella arizonae in sheep. Can Vet J 31: 42.
68. Manolis SC, Webb GJ, Pinch D, Melville L, Hollis G (1991) Salmonella in captive crocodiles (Crocodylus johnstoni and C. porosus). Aust Vet J 68: 102-105.
69. Kenny DE, Baier J, Getzy DM (1997) Salmonellosis in captive black rhinoceroses (Diceros bicornis). J Zoo Wildl Med 28: 307-311.
70. Macri NP, Stevenson GW, Wu CC (1997) Salmonella arizonae sepsis in a lynx. J Wildl Dis 33: 908-911.
71. Oros J, Rodriguez JL, Fernandez A, Herraez P, Espinosa de los Monteros A, et al. (1998) Simultaneous occurrence of Salmonella arizonae in a sulfur crested cockatoo (Cacatua galerita galerita) and iguanas. Avian Dis 42: 818-823.
72. Briones V, Tellez S, Goyache J, Ballesteros C, del Pilar Lanzarot M, et al. (2004) Salmonella diversity associated with wild reptiles and amphibians in Spain. Environ Microbiol 6: 868-871.
73. Oloya J, Doetkott D, Khaitsa ML (2009) Antimicrobial drug resistance and molecular characterization of Salmonella isolated from domestic animals, humans, and meat products. Foodborne Pathog Dis 6: 273-284.
74. Foley SL, Lynne AM (2008) Food animal-associated Salmonella challenges: pathogenicity and antimicrobial resistance. J Anim Sci 86: E173-187.
75. Devi SJ, Murray CJ (1991) Cockroaches (Blatta and Periplaneta species) as reservoirs of drug-resistant salmonellas. Epidemiol Infect 107: 357-361.
76. Feeley JC, Treger MD (1969) Penetration of turtle eggs by Salmonella braenderup. Public Health Rep 84: 156-158.
77. Gopee NV, Adesiyun AA, Caesar K (2000) Retrospective and longitudinal study of salmonellosis in captive wildlife in Trinidad. J Wildl Dis 36: 284-293.
78. Sojka WJ, Wray C, Shreeve J, Benson AJ (1977) Incidence of salmonella infection in animals in England and Wales 1968--1974. J Hyg (Lond) 78: 43-56.
79. Kikuvi GM, Ombui JN, Mitema ES, Schwarz S (2007) Antimicrobial resistance in Salmonella serotypes isolated from slaughter animals in Kenya. East Afr Med J 84: 233-239.
80. Foley SL, Lynne AM, Nayak R (2008) Salmonella challenges: prevalence in swine and poultry and potential pathogenicity of such isolates. J Anim Sci 86: E149-162.
81. Pourciau SS, Springer WT (1978) Frequency and duration of paratyphoid organism shedding by experimentally infected bobwhite quail (Colinus virginianus). J Wildl Dis 14: 203-207.
82. Bangert RL, Ward AC, Stauber EH, Cho BR, Widders PR (1988) A survey of the aerobic bacteria in the feces of captive raptors. Avian Dis 32: 53-62.
83. Jijon S, Wetzel A, LeJeune J (2007) Salmonella enterica isolated from wildlife at two Ohio rehabilitation centers. J Zoo Wildl Med 38: 409-413.
84. Chong Y, Kwon OH, Lee SY, Chung KS, Shimada T (1991) Salmonella enterica subspecies diarizonae bacteremia in an infant with enteritis--a case report. Yonsei Med J 32: 275-278.
85. Madsen M, Hangartner P, West K, Kelly P (1998) Recovery rates, serotypes, and antimicrobial susceptibility patterns of salmonellae isolated from cloacal swabs of wild Nile crocodiles (Crocodylus niloticus) in Zimbabwe. J Zoo Wildl Med 29: 31-34.
86. Schroter M, Roggentin P, Hofmann J, Speicher A, Laufs R, et al. (2004) Pet snakes as a reservoir for Salmonella enterica subsp. diarizonae (Serogroup IIIb): a prospective study. Appl Environ Microbiol 70: 613-615.
87. Ferreras Mdel C, Munioz M, Perez V, Benavides J, Garcia-Pariente C, et al. (2007) Unilateral orchitis and epididymitis caused by Salmonella enterica subspecies diarizonae infection in a ram. J Vet Diagn Invest 19: 194-197.
88. Sechter I, Katzenelson E, Reisfeld A (1991) Salmonella serovars (others than Typhi and Paratyphi) from extra-intestinal sources. Israel, 1984-9. Epidemiol Infect 106: 485-488.
89. Morse EV, Duncan MA, Estep DA, Riggs WA, Blackburn BO (1976) Canine salmonellosis: A review and report of dog to child transmission of Salmonella enteritidis. Am J Public Health 66: 82-84.
90. Metz H, Lieb U (1980) [Enteritis salmonellae in man and animal from 1953 to 1975 in Southern Bavaria (author's transl)]. Zentralbl Bakteriol Mikrobiol Hyg B 171: 231-255.
91. Silva CA, Blondel CJ, Quezada CP, Porwollik S, Andrews-Polymenis HL, et al. Infection of mice by Salmonella enterica serovar Enteritidis involves additional genes that are absent in the genome of serovar Typhimurium. Infect Immun.
92. Quiroz TS, Nieto PA, Tobar HE, Salazar-Echegarai FJ, Lizana RJ, et al. Excision of an Unstable Pathogenicity Island in Salmonella enterica Serovar Enteritidis Is Induced during Infection of Phagocytic Cells. PLoS One 6: e26031.
93. Van Immerseel F, Pasmans F, De Buck J, Rychlik I, Hradecka H, et al. (2004) Cats as a risk for transmission of antimicrobial drug-resistant Salmonella. Emerg Infect Dis 10: 2169-2174.
94. Fox JG, Beaucage CM (1979) The incidence of Salmonella in random-source cats purchased for use in research. J Infect Dis 139: 362-365.
95. Thompson H, Wright NG (1969) Canine salmonellosis. J Small Anim Pract 10: 579-582.
96. Trawinska B, Saba L, Wdowiak L, Ondrasovicova O, Nowakowicz-Debek B (2008) Evaluation of Salmonella rod incidence in poultry in the Lublin Province over the years 2001-2005. Ann Agric Environ Med 15: 131-134.
97. Guerin MT, Martin SW, Darlington GA, Rajic A (2005) A temporal study of Salmonella serovars in animals in Alberta between 1990 and 2001. Can J Vet Res 69: 88-99.
98. Steffen EK, Wagner JE (1983) Salmonella enteriditis serotype Amsterdam in a commercial rat colony. Lab Anim Sci 33: 454-456.
99. Lapuz R, Tani H, Sasai K, Shirota K, Katoh H, et al. (2008) The role of roof rats ( Rattus rattus) in the spread of Salmonella Enteritidis and S. Infantis contamination in layer farms in eastern Japan. Epidemiol Infect 136: 1235-1243.
100. Richardson A (1975) Outbreaks of bovine salmonellosis caused by serotypes other than S. dublin and S. typhimurium. J Hyg (Lond) 74: 195-203.
101. van Duijkeren E, van Klingeren B, Vulto AG, Sloet van Oldruitenborgh-Oosterbaan MM, Breukink HJ, et al. (1995) In vitro susceptibility to antimicrobial drugs of 62 Salmonella strains isolated from horses in The Netherlands. Vet Microbiol 45: 19-26.
102. Renter DG, Gnad DP, Sargeant JM, Hygnstrom SE (2006) Prevalence and serovars of Salmonella in the feces of free-ranging white-tailed deer (Odocoileus virginianus) in Nebraska. J Wildl Dis 42: 699-703.
103. Pedersen K, Hansen HC, Jorgensen JC, Borck B (2002) Serovars of Salmonella isolated from Danish turkeys between 1995 and 2000 and their antimicrobial resistance. Vet Rec 150: 471-474.
104. Williams DR, Bellhouse R (1974) The prevalence of salmonellas in mink. J Hyg (Lond) 72: 71-78.
105. Olsen AR, Hammack TS (2000) Isolation of Salmonella spp. from the housefly, Musca domestica L., and the dump fly, Hydrotaea aenescens (Wiedemann) (Diptera: Muscidae), at caged-layer houses. J Food Prot 63: 958-960.
106. Carrasco SE, Burek KA, Beckmen KB, Oaks JL, Davis MA, et al. Aerobic oral and rectal bacteria of free-ranging steller sea lion pups and juveniles (eumetopias jubatus) in alaska. J Wildl Dis 47: 807-820.
107. Ma JS, Chen PY, Lau YJ, Chi CS (2003) Brain abscess caused by Salmonella enterica subspecies houtenae in a patient with chronic granulomatous disease. J Microbiol Immunol Infect 36: 282-284.
108. Wybo I, Potters D, Plaskie K, Covens L, Collard JM, et al. (2004) Salmonella enterica subspecies houtenae serotype 44:z4, z23:--as a rare cause of meningitis. Acta Clin Belg 59: 232-234.
109. Tabarani CM, Bennett NJ, Kiska DL, Riddell SW, Botash AS, et al. Empyema of preexisting subdural hemorrhage caused by a rare salmonella species after exposure to bearded dragons in a foster home. J Pediatr 156: 322-323.
110. Woodward DL, Khakhria R, Johnson WM (1997) Human salmonellosis associated with exotic pets. J Clin M10robiol 35: 2786-2790.
111. Phillips WE Jr., Hatkin JM (1978) Isolation of Salmonella houtenae from a cockateel. Avian Dis 22: 350-353.
112. Runkel NS, Rodriguez LF, Moody FG, LaRocco MT, Blasdel T (1991) Salmonella infection of the biliary and intestinal tract of wild opossums. Lab Anim Sci 41: 54-56.
113. Snehalatha S, Mathai E, Jayasheela M, Chandy M, Lalitha MK, et al. (1992) Salmonella choleraesuis subsp. indica serovar bornheim causing urinary tract infection. J Clin Microbiol 30: 2504-2505.
114. Kohler PF (1964) Hospital Salmonellosis. a Report of 23 Cases of Gastroenteritis Caused by Salmonella Infantis. Jama 189: 6-10.
115. Magistrali C, Dionisi AM, De Curtis P, Cucco L, Vischi O, et al. (2008) Contamination of Salmonella spp. in a pig finishing herd, from the arrival of the animals to the slaughterhouse. Res Vet Sci 85: 204-207.
116. Loynachan AT, Nugent JM, Erdman MM, Harris DL (2004) Acute infection of swine by various Salmonella serovars. J Food Prot 67: 1484-1488.
117. Jayarao BM, Biro G, Kovacs S, Domjan H, Fabian A (1989) Prevalence of Salmonella serotypes in pigs and evaluation of a rapid, presumptive test for detection of Salmonella in pig faeces. Acta Vet Hung 37: 39-44.
118. Mortelmans J, Huygelen C, Pinckers F (1958) Isolation of Salmonella infantis from an aborted bovine foetus. Nature 181: 1539-1540.
119. Tavechio AT, Ghilardi AC, Peresi JT, Fuzihara TO, Yonamine EK, et al. (2002) Salmonella serotypes isolated from nonhuman sources in Sao Paulo, Brazil, from 1996 through 2000. J Food Prot 65: 1041-1044.
120. Bhatia TR, McNabb GD (1980) Dissemination of Salmonella in broiler-chicken operations. Avian Dis 24: 616-624.
121. Roy P, Dhillon AS, Lauerman LH, Schaberg DM, Bandli D, et al. (2002) Results of salmonella isolation from poultry products, poultry, poultry environment, and other characteristics. Avian Dis 46: 17-24.
122. Smith BP, Reina-Guerra M, Hardy AJ (1978) Prevalence and epizootiology of equine salmonellosis. J Am Vet Med Assoc 172: 353-356.
123. Gordon RF, Tucker JF (1957) The isolation of Salmonella infantis from a turkey poult. Mon Bull Minist Health Public Health Lab Serv 16: 71.
124. Bartlett KH, Trust TJ (1976) Isolation of salmonellae and other potential pathogens from the freshwater aquarium snail Ampullaria. Appl Environ Microbiol 31: 635-639.
125. White FH, Forrester DJ (1979) Antimicrobial resistant Salmonella spp. isolated from double-crested cormorants (Phalacrocorax auritus) and common loons (Gavia immer) in Florida. J Wildl Dis 15: 235-237.
126. Sato Y, Kuwamoto R (1999) A case of canine salmonellosis due to Salmonella infantis. J Vet Med Sci 61: 71-72.
127. Selander RK, Beltran P, Smith NH, Helmuth R, Rubin FA, et al. (1990) Evolutionary genetic relationships of clones of Salmonella serovars that cause human typhoid and other enteric fevers. Infect Immun 58: 2262-2275.
128. Adesiyun AA, Caesar K, Inder L (1998) Prevalence of Salmonella and Campylobacter species in animals at Emperor Valley Zoo, Trinidad. J Zoo Wildl Med 29: 237-239.
129. Gordon HS, Hoffman SJ, et al. (1949) Serous arthritis of the knee joint; report of a case caused by Salmonella typhosa and Salmonella montevideo in a child. J Am Med Assoc 141: 460.
130. Gronroos JA (1953) Infections caused by Salmonella montevideo and the bacteriological identification of this strain. Ann Med Exp Biol Fenn 31: 339-347.
131. Ellenbogen NC, Raim J, Grossman L (1955) Salmonella sp. (type Montevideo) osteomyelitis; report of a case and review of the literature. AMA Am J Dis Child 90: 275-279.
132. Fedorka-Cray PJ, Dargatz DA, Thomas LA, Gray JT (1998) Survey of Salmonella serotypes in feedlot cattle. J Food Prot 61: 525-530.
133. Sato K, Carpenter TE, Case JT, Walker RL (2001) Spatial and temporal clustering of Salmonella serotypes isolated from adult diarrheic dairy cattle in California. J Vet Diagn Invest 13: 206-212.
134. Simmons DJ, Simpson W (1980) Salmonella montevideo salmonellosis in laboratory mice: successful treatment of the disease by oral oxytetracycline. Lab Anim 14: 217-219.
135. Sharp JC, Reilly WJ, Linklater KA, Inglis DM, Johnston WS, et al. (1983) Salmonella montevideo infection in sheep and cattle in Scotland, 1970-81. J Hyg (Lond) 90: 225-232.
136. Linklater KA (1983) Abortion in sheep associated with Salmonella montevideo infection. Vet Rec 112: 372-374.
137. Kirk JH, Holmberg CA, Jeffrey JS (2002) Prevalence of Salmonella spp in selected birds captured on California dairies. J Am Vet Med Assoc 220: 359-362.
138. Trudel F (1947) Salmonella montevideo Infection. Can J Comp Med Vet Sci 11: 245-246.
139. Coulson JC, Butterfield J, Thomas C (1983) The herring gull Larus argentatus as a likely transmitting agent of Salmonella montevideo to sheep and cattle. J Hyg (Lond) 91: 437-443.
140. Fonseca RJ, Dubey LM (1994) Salmonella montevideo sepsis from a pet snake. Pediatr Infect Dis J 13: 550.
141. Caldow GL, Graham MM (1998) Abortion in foxhounds and a ewe flock associated with Salmonella montevideo infection. Vet Rec 142: 138-139.
142. Schotte U, Borchers D, Wulff C, Geue L (2007) Salmonella Montevideo outbreak in military kennel dogs caused by contaminated commercial feed, which was only recognized through monitoring. Vet Microbiol 119: 316-323.
143. Nesse LL, Refsum T, Heir E, Nordby K, Vardund T, et al. (2005) Molecular epidemiology of Salmonella spp. isolates from gulls, fish-meal factories, feed factories, animals and humans in Norway based on pulsed-field gel electrophoresis. Epidemiol Infect 133: 53-58.
144. Nesse LL, Lovold T, Bergsjo B, Nordby K, Wallace C, et al. (2005) Persistence of orally administered Salmonella enterica serovars Agona and Montevideo in Atlantic salmon (Salmo salar L.). J Food Prot 68: 1336-1339.
145. Mills KL (1964) Osteomyelitis of the Spine Due to Salmonella Muenchen. J Bone Joint Surg Br 46: 697-699.
146. Samuel JL, O'Boyle DA, Mathers WJ, Frost AJ (1980) Isolation of Salmonella from mesenteric lymph nodes of healthy cattle at slaughter. Res Vet Sci 28: 238-241.
147. Gebreyes WA, Thakur S (2005) Multidrug-resistant Salmonella enterica serovar Muenchen from pigs and humans and potential interserovar transfer of antimicrobial resistance. Antimicrob Agents Chemother 49: 503-511.
148. Kodjo A, Villard L, Prave M, Ray S, Grezel D, et al. (1997) Isolation and identification of Salmonella species from chelonians using combined selective media, serotyping and ribotyping. Zentralbl Veterinarmed B 44: 625-629.
149. Pasmans F, De Herdt P, Dewulf J, Haesebrouck F (2002) Pathogenesis of infections with Salmonella enterica subsp. enterica serovar Muenchen in the turtle Trachemys scripta scripta. Vet Microbiol 87: 315-325.
150. McOrist S, Miller GT (1981) Salmonellosis in transported feral goats. Aust Vet J 57: 389-390.
151. Venter EH, van Vuuren M, Carstens J, van der Walt ML, Nieuwoudt B, et al. (2003) A molecular epidemiologic investigation of Salmonella from a meat source to the feces of captive cheetah (Acinonyx jubatus). J Zoo Wildl Med 34: 76-81.
152. Potter AS, Reid SA, Fenwick SG Prevalence of salmonella in fecal samples of Western grey kangaroos (macropus fuliginosus). J Wildl Dis 47: 880-887.
153. Bezanson GS, Khakhria R, Pagnutti D (1983) Plasmid profiles of value in differentiating Salmonella muenster isolates. J Clin Microbiol 17: 1159-1160.
154. Vigo GB, Cappuccio JA, Pineyro PE, Salve A, Machuca MA, et al. (2009) Salmonella enterica subclinical infection: bacteriological, serological, pulsed-field gel electrophoresis, and antimicrobial resistance profiles--longitudinal study in a three-site farrow-to-finish farm. Foodborne Pathog Dis 6: 965-972.
155. Sanford SE (1984) Some respiratory and enteric diseases of cattle: an update. Mod Vet Pract 65: 265-268.
156. Nayak R, Stewart-King T (2008) Molecular epidemiological analysis and microbial source tracking of Salmonella enterica serovars in a preharvest turkey production environment. Foodborne Pathog Dis 5: 115-126.
157. Fairbrother RW (1947) A case of enteric fever caused by Salm. paratyphi A. Mon Bull Minist Health Public Health Lab Serv 6: 180-182.
158. Freerksen E, Rosenfeld M, Freerksen R, Kruger-Thiemer M (1977) Treatment of chronic salmonella carriers. Study with 40 cases of S. typhi, 19 cases of S. paratyphi b and 28 cases of S. enteritidis strains. Chemotherapy 23: 192-210.
159. George JT, Wallace JG, Morrison HR, Harbourne JF (1972) Paratyphoid in man and cattle. Br Med J 3: 208-211.
160. Roland KL, Tinge SA, Kochi SK, Thomas LJ, Killeen KP Reactogenicity and immunogenicity of live attenuated Salmonella enterica serovar Paratyphi A enteric fever vaccine candidates. Vaccine 28: 3679-3687.
161. Mikhail IA, Higashi GI, Edman DC, Elwan SH (1982) Interaction of Salmonella paratyphi A and Schistosoma mansoni in hamsters. Am J Trop Med Hyg 31: 328-334.
162. Poon MC, Sanders MG (1972) Hepatic abscess caused by Salmonella paratyphi B. Can Med Assoc J 107: 529-531.
163. Broadhead RL, Sehgal KC (1981) Necrotising enterocolitis associated with Salmonella paratyphi B type 4, 5. Ann Trop Paediatr 1: 65-68.
164. Caglar MK, Yalaz K, Caglar M, Yilgor E (1986) Lobar pneumonia and pneumatocele formation due to Salmonella paratyphi B in an infant. Turk J Pediatr 28: 279-283.
165. Spence JA, Mogere R, Palmer TJ, Rowe PH (1987) Severe rectal bleeding due to Salmonella paratyphi B. Br Med J (Clin Res Ed) 294: 1589.
166. Gray RD, Harvey SM (1955) An outbreak of infection in a children's home by an unusual member of the Salmonella group related to Salm. paratyphi B. Mon Bull Minist Health Public Health Lab Serv 14: 74-78.
167. Singh BR, Jyoti J, Chandra M, Babu N, Sharma G (2009) Drug resistance patterns of Salmonella isolates of equine origin from India. J Infect Dev Ctries 3: 141-147.
168. Carter PB, Collins FM (1974) Growth of typhoid and paratyphoid bacilli in intravenously infected mice. Infect Immun 10: 816-822.
169. Harbourne JF, Randall CJ, Luery KW, Wallace JG (1972) Salmonella paratyphi B infection in dairy cows. I. Vet Rec 91: 112-114.
170. Thomas GW, Harbourne JF (1972) Salmonella paratyphi B infection in dairy cows. II. Investigation of an active carrier. Vet Rec 91: 148-150.
171. Thomas GW (1978) Salmonella paratyphi B in cattle. Vet Rec 103: 512.
172. Van Immerseel F, Meulemans L, De Buck J, Pasmans F, Velge P, et al. (2004) Bacteria-host interactions of Salmonella Paratyphi B dT+ in poultry. Epidemiol Infect 132: 239-243.
173. Rodriguez I, Barownick W, Helmuth R, Mendoza MC, Rodicio MR, et al. (2009) Extended-spectrum {beta}-lactamases and AmpC {beta}-lactamases in ceftiofur-resistant Salmonella enterica isolates from food and livestock obtained in Germany during 2003-07. J Antimicrob Chemother 64: 301-309.
174. Nagano N, Oana S, Nagano Y, Arakawa Y (2006) A severe Salmonella enterica serotype Paratyphi B infection in a child related to a pet turtle, Trachemys scripta elegans. Jpn J Infect Dis 59: 132-134.
175. Hazeleger WC, Bolder NM, Beumer RR, Jacobs-Reitsma WF (2008) Darkling beetles (Alphitobius diaperinus) and their larvae as potential vectors for the transfer of Campylobacter jejuni and Salmonella enterica serovar paratyphi B variant Java between successive broiler flocks. Appl Environ Microbiol 74: 6887-6891.
176. Saha SN, Khanna PN, Gupta BR, Varma KC (1981) Isolation of Salmonella paratyphi B var odense in India. Indian J Public Health 25: 85-86.
177. Kumar AA, Mallick BB, Uppal PK (1979) Isolation of Salmonella paratyphi B and Salmonella typhimurium from guineapigs. Indian J Med Res 69: 390-392.
178. Carter HS, Weir IB (1952) A dog as a probable source of human infection with Salmonella paratyphi-B. J Pathol Bacteriol 64: 230-232.
179. Jacobs MR, Koornhof HJ, Crisp SI, Palmhert HL, Fitzstephens A (1978) Enteric fever caused by Salmonella paratyphi C in South and South West Africa. S Afr Med J 54: 434-438.
180. Freidin N, Merzbach D, Kleinhaus U, Eidelman S (1985) Recurrent intraabdominal abscess caused by Salmonella paratyphi C. J Clin Microbiol 21: 284-285.
181. Lang R, Maayan MC, Lidor C, Savin H, Kolman S, et al. (1992) Salmonella paratyphi C osteomyelitis: report of two separate episodes 17 years apart. Scand J Infect Dis 24: 793-796.
182. Bolivar R, Bodey GP, Velasquez WS (1982) Recurrent Salmonella meningitis in a compromised host. Cancer 50: 2034-2036.
183. (1991) Multistate outbreak of Salmonella poona infections--United States and Canada, 1991. MMWR Morb Mortal Wkly Rep 40: 549-552.
184. Wu CC, de Gortari MJ, Lin TL, Barrett B (1998) Ribotyping of Salmonella poona in iguana-associated zoonotic salmonellosis. J Vet Diagn Invest 10: 188-190.
185. Falade S (1976) Isolation of Salmonella poona from diarrhoeic Nigerian goats. Vet Rec 99: 419.
186. Molla W, Molla B, Alemayehu D, Muckle A, Cole L, et al. (2006) Occurrence and antimicrobial resistance of Salmonella serovars in apparently healthy slaughtered sheep and goats of central Ethiopia. Trop Anim Health Prod 38: 455-462.
187. Oboegbulem SI, Okoronkwo I (1990) Salmonellae in the African great cane rat (Thryonomys swinderianus). J Wildl Dis 26: 119-121.
188. Caldwell KN, Adler BB, Anderson GL, Williams PL, Beuchat LR (2003) Ingestion of Salmonella enterica serotype Poona by a free-living mematode, Caenorhabditis elegans, and protection against inactivation by produce sanitizers. Appl Environ Microbiol 69: 4103-4110.
189. Kenney SJ, Anderson GL, Williams PL, Millner PD, Beuchat LR (2005) Persistence of Escherichia coli O157:H7, Salmonella Newport, and Salmonella Poona in the gut of a free-living nematode, Caenorhabditis elegans, and transmission to progeny and uninfected nematodes. Int J Food Microbiol 101: 227-236.
190. Sandberg M, Alvseike O, Skjerve E (2002) The prevalence and dynamics of Salmonella enterica IIIb 61:k:1,5,(7) in sheep flocks in Norway. Prev Vet Med 52: 267-275.
191. Hidalgo-Vila J, Diaz-Paniagua C, de Frutos-Escobar C, Jimenez-Martinez C, Perez-Santigosa N (2007) Salmonella in free living terrestrial and aquatic turtles. Vet Microbiol 119: 311-315.
192. Edwards PR, Moran AB (1945) Salmonella Cultures Which Resemble the Sendai Type. J Bacteriol 50: 257-260.
193. McFadzean AJ, Huang CT (1952) Bacterial endocarditis due to Salmonella sp. (Type Sendai) with recovery. AMA Arch Intern Med 90: 858-862.
194. Saha MR, Saha D, Dutta P, Mitra U, Bhattacharya SK (2001) Isolation of Salmonella enterica serotypes from children with diarrhoea in Calcutta, India. J Health Popul Nutr 19: 301-305.
195. Dorn-In S, Fries R, Padungtod P, Kyule MN, Baumann MP, et al. (2009) A cross-sectional study of Salmonella in pre-slaughter pigs in a production compartment of northern Thailand. Prev Vet Med 88: 15-23.
196. Fenlon DR (1983) A comparison of salmonella serotypes found in the faeces of gulls feeding at a sewage works with serotypes present in the sewage. J Hyg (Lond) 91: 47-52.
197. Takasaka M, Kohno A, Sakakibara I, Narita H, Honjo S (1988) An outbreak of salmonellosis in newly imported cynomolgus monkeys. Jpn J Med Sci Biol 41: 1-13.
198. Dahshan H, Shahada F, Chuma T, Moriki H, Okamoto K Genetic analysis of multidrug-resistant Salmonella enterica serovars Stanley and Typhimurium from cattle. Vet Microbiol.
199. Scott WM (1926) The "Thompson" Type of Salmonella. J Hyg (Lond) 25: 398-405.
200. Gordon RF, Buxton A (1945) The isolation of Salmonella thompson from outbreaks of disease in chicks. J Hyg (Lond) 44: 179-183.
201. Chisholm SA, Crichton PB, Knight HI, Old DC (1999) Molecular typing of Salmonella serotype Thompson strains isolated from human and animal sources. Epidemiol Infect 122: 33-39.
202. Sternberg S, Johnsson A, Aspan A, Bergstrom K, Kallay TB, et al. (2008) Outbreak of Salmonella Thompson infection in a Swedish dairy herd. Vet Rec 163: 596-599.
203. Buxton A, Gordon RF (1947) The epidemiology and control of Salmonella thompson infection of fowls. J Hyg (Lond) 45: 265-281.
204. Kirkpatrick CE, Colvin BA (1986) Salmonella spp. in nestling common barn-owls (Tyto alba) from southwestern New Jersey. J Wildl Dis 22: 340-343.
205. Gaertner JP, Hahn D, Rose FL, Forstner MR (2008) Detection of salmonellae in different turtle species within a headwater spring ecosystem. J Wildl Dis 44: 519-526.
206. Vogelsang TM, Boe J (1948) Temporary and chronic carriers of Salmonella typhi and Salmonella paratyphi B. J Hyg (Lond) 46: 252-261.
207. Daigle F (2008) Typhi genes expressed during infection or involved in pathogenesis. J Infect Dev Ctries 2: 431-437.
208. Lucas RL, Lee CA (2000) Unravelling the mysteries of virulence gene regulation in Salmonella typhimurium. Mol Microbiol 36: 1024-1033.
209. Kastello MD, Spertzel RO (1973) The Rhesus monkey as a model for the study of infectious disease. Am J Phys Anthropol 38: 501-504.
210. Barber C, Eylan E (1975) Confirmation of the protective role of proteins from S. typhimurium in infection of mice with their natural pathogen. Zentralbl Bakteriol Orig A 230: 461-465.
211. Lawley TD, Chan K, Thompson LJ, Kim CC, Govoni GR, et al. (2006) Genome-wide screen for Salmonella genes required for long-term systemic infection of the mouse. PLoS Pathog 2: e11.
212. Schott HC, 2nd, Ewart SL, Walker RD, Dwyer RM, Dietrich S, et al. (2001) An outbreak of salmonellosis among horses at a veterinary teaching hospital. J Am Vet Med Assoc 218: 1152-1159, 1100.
213. Bartlett KH, Trust TJ, Lior H (1977) Small pet aquarium frogs as a source of Salmonella. Appl Environ Microbiol 33: 1026-1029.
214. Madewell BR, McChesney AE (1975) Salmonellosis in a human infant, a cat, and two parakeets in the same household. J Am Vet Med Assoc 167: 1089-1090.
215. Cooper BS, MacFarlane DJ (1974) Single or double vaccination schedules in sheep against experimental infection with Salmonella typhimurium or Salmonella bovismorbificans. N Z Vet J 22: 95-99.
216. Gough J, McEwen B (2000) Salmonella typhimurium DT 104 in sheep. Can Vet J 41: 413.
217. Elissalde MH, Ziprin RL, Huff WE, Kubena LF, Harvey RB (1994) Effect of ochratoxin A on Salmonella-challenged broiler chicks. Poult Sci 73: 1241-1248.
218. Bottjer KP, Hirst DC, Slonka GF (1978) Nematospiroides dubius as a vector for Salmonella typhimurium. Am J Vet Res 39: 151-153.
219. Betancor L, Pereira M, Martinez A, Giossa G, Fookes M, et al. Prevalence of Salmonella enterica in poultry and eggs in Uruguay during an epidemic due to Salmonella enterica serovar Enteritidis. J Clin Microbiol 48: 2413-2423.
220. Watson PR, Galyov EE, Paulin SM, Jones PW, Wallis TS (1998) Mutation of invH, but not stn, reduces Salmonella-induced enteritis in cattle. Infect Immun 66: 1432-1438.
221. Lucas B, Bumann D, Walduck A, Koesling J, Develioglu L, et al. (2001) Adoptive transfer of CD4+ T cells specific for subunit A of Helicobacter pylori urease reduces H. pylori stomach colonization in mice in the absence of interleukin-4 (IL-4)/IL-13 receptor signaling. Infect Immun 69: 1714-1721.
222. Adaska JM, Silva AJ, Sischo WM (2008) Comparison of Salmonella enterica subspecies enterica serovar Typhimurium isolates from dairy cattle and humans using in vitro assays of virulence. Vet Microbiol 128: 90-95.
223. Labrousse A, Chauvet S, Couillault C, Kurz CL, Ewbank JJ (2000) Caenorhabditis elegans is a model host for Salmonella typhimurium. Curr Biol 10: 1543-1545.
224. Oza JP, Yeh JB, Reich NO (2005) DNA methylation modulates Salmonella enterica serovar Typhimurium virulence in Caenorhabditis elegans. FEMS Microbiol Lett 245: 53-59.
225. Rahman H, Deka PJ, Chakraborty A, Narayan G (2005) Salmonellosis in pigmy hogs (Sus salvanius)--a critically endangered species of mammal. Rev Sci Tech 24: 959-964.
226. Belfort R Jr., Toledo MR, Burnier M, Smith RL, Silva VL, et al. (1985) Experimental guinea pig ocular infection by Salmonella typhimurium. Invest Ophthalmol Vis Sci 26: 591-594.
227. Brunsson I (1987) Enteric nerves mediate the fluid secretory response due to Salmonella typhimurium R5 infection in the rat small intestine. Acta Physiol Scand 131: 609-617.
228. Sato Y, Wada K (1995) Isolation of Salmonella typhimurium from zebra finches (Poephila guttata). J Vet Med Sci 57: 137-138.
229. Prescott JF, Poppe C, Goltz J, Campbell GD (1998) Salmonella typhimurium phage type 40 in feeder birds. Vet Rec 142: 732.
230. Helm JD, Hines RK, Hill JE, Caver JA (1999) Multiple drug-resistant Salmonella typhimurium DT104 and DT104b isolated in bobwhite quail (Colinus virginianus). Avian Dis 43: 788-791.
231. Sato Y, Kobayash C, Ichikawa K, Kuwamoto R, Matsuura S, et al. (2000) An occurrence of Salmonella typhimurium infection in sika deer (Cervus nippon). J Vet Med Sci 62: 313-315.
232. Verwoerd DJ (2000) Ostrich diseases. Rev Sci Tech 19: 638-661.
233. Handeland K, Refsum T, Johansen BS, Holstad G, Knutsen G, et al. (2002) Prevalence of Salmonella typhimurium infection in Norwegian hedgehog populations associated with two human disease outbreaks. Epidemiol Infect 128: 523-527.
234. Smith KE, Anderson F, Medus C, Leano F, Adams J (2005) Outbreaks of salmonellosis at elementary schools associated with dissection of owl pellets. Vector Borne Zoonotic Dis 5: 133-136.
235. Handeland K, Nesse LL, Lillehaug A, Vikoren T, Djonne B, et al. (2008) Natural and experimental Salmonella Typhimurium infections in foxes (Vulpes vulpes). Vet Microbiol 132: 129-134.
